# Antenatal influenza and pertussis vaccination in Western Australia: a cross-sectional survey of vaccine uptake and influencing factors

**DOI:** 10.1186/s12884-018-2051-3

**Published:** 2018-10-24

**Authors:** Donna B. Mak, Annette K. Regan, Dieu T. Vo, Paul V. Effler

**Affiliations:** 1 Communicable Disease Control Directorate, Department of Health, Shenton Park, Western Australia; 2 School of Medicine, University of Notre Dame, Fremantle, Western Australia; 3 School of Public Health, Curtin University, Bentley, Western Australia; 4 School of Population Health, University of Western Australia, Crawley, Western Australia; 5 School of Pathology and Laboratory Medicine, University of Western Australia, Crawley, Western Australia

**Keywords:** Maternal health, Vaccination, Influenza vaccine, Pertussis vaccine, Antenatal care

## Abstract

**Background:**

Influenza and pertussis vaccines have been recommended in Australia for women during each pregnancy since 2010 and 2015, respectively. Estimating vaccination coverage and identifying factors affecting uptake are important for improving antenatal immunisation services.

**Methods:**

A random sample of 800 Western Australian women ≥18 years of age who gave birth between 4th April and 4th October 2015 were selected. Of the 454 (57%) who were contactable by telephone, 424 (93%) completed a survey. Data were weighted by maternal age and area of residence to ensure representativeness. The proportion immunised against influenza and pertussis was the main outcome measure; multivariate logistic regression was used to identify factors significantly associated with antenatal vaccination. Results from the 2015 study were compared to similar surveys conducted in 2012–2014.

**Results:**

In 2015, 71% (95% CI 66–75) of women received pertussis-containing vaccine and 61% (95% CI 56–66) received influenza vaccine during pregnancy; antenatal influenza vaccine coverage was 18% higher than in 2014 (43%; 95% CI: 34–46). Pertussis and influenza vaccine were co-administered for 68% of the women who received both vaccines. The majority of influenza vaccinations in 2015 were administered during the third trimester of pregnancy, instead of the second trimester, as was observed in prior years. Women whose care provider recommended both antenatal vaccinations had significantly higher odds of being vaccinated against both influenza and pertussis (OR 33.3, 95% CI: 15.15–73.38). Of unvaccinated mothers, 53.6% (95% CI: 45.9–61.3) and 78.3% (95% CI: 70.4–85.3) reported that they would have been vaccinated against influenza and pertussis, respectively, if their antenatal care provider had recommended it.

**Conclusions:**

Pertussis vaccination coverage was high in the first year of an antenatal immunisation program in Western Australia. Despite a substantial increase in influenza vaccination uptake between 2014 and 2015, coverage remained below that for pertussis. Our data suggest influenza and pertussis vaccination rates of 83% and 94%, respectively, are achievable if providers were to recommend them to all pregnant women.

## Background

Antenatal influenza vaccination can protect pregnant women from serious complications of influenza and prevent severe, potentially fatal influenza and pertussis infections in young infants through maternal antibody transfer [1]. Vaccinating pregnant women for pertussis during the third trimester of pregnancy ensures maximum transfer of maternal antibodies from the vaccine to the child through the placental membrane, thereby protecting young infants from the life-threatening complications of pertussis [2, 3]. In Australia, antenatal influenza vaccination has been recommended to pregnant women at any trimester during their pregnancy during the flu season and funded through the national immunisation program since 2010 [4]. Acellular pertussis-diphtheria-tetanus vaccine has been recommended during the third trimester of every pregnancy since 2015 [5]. The Western Australian (WA) government has funded provision of antenatal pertussis vaccination since March 2015, following the death of a one-month old infant from pertussis [6, 7].

Despite the availability of free vaccine and the demonstrated effectiveness in pregnant mothers and infants under 6 months of age [8, 9], previous research has shown uptake of influenza vaccine during pregnancy to be sub-optimal [10]. A study in 2014 found that just 41% of pregnant women in WA received influenza vaccine during pregnancy [8] with lack of recommendation of the vaccine from health providers being a main barrier for uptake of the antenatal flu vaccine [10]. More than 40% of women were not recommended the vaccine during pregnancy. The majority of women reported that they would have been vaccinated if a healthcare professional had recommended the vaccine to them. The study also found that many women were vaccinated to protect their unborn child suggesting that promotional efforts should emphasize on the importance of the vaccine for the child [10]. A systematic review has also identified inadequate knowledge of influenza risk and concerns about the safety of the antenatal influenza as barriers to uptake [11].

As the introduction of the antenatal pertussis vaccination program is relatively recent compared to the antenatal influenza vaccination program, data on pertussis coverage in Western Australia is limited. However, factors influencing uptake of the antenatal pertussis vaccine in other Australian states have been documented. A 2016 survey of 136 Victorian pregnant women found recommendation of the pertussis vaccine by a health care provider and belief in protection for the unborn child against pertussis was a main determinant of vaccine uptake [12]. The importance of health care provider recommendation was also demonstrated in surveys of Aboriginal mothers in Western Australia [13] and women from culturally and linguistically diverse backgrounds in Melbourne [14].

The aim of this study was to: 1) measure influenza and pertussis vaccine coverage during pregnancy in the first year after introduction of an antenatal pertussis vaccination program; and 2) compare factors associated with the uptake of each vaccine.

## Methods

Annual surveys of antenatal influenza vaccination uptake have been conducted by the WA Department of Health (WA DOH) since 2012 [10], and in 2015, this survey was expanded to include pertussis vaccination. A sample of women ≥18 years who had given birth to a live infant between 4th April and 4th October 2015 (i.e. the period when the 2015 seasonal influenza vaccine was readily available) were randomly selected from the state’s perinatal birth dataset; the Midwives Notification System (MNS) is a mandatory data reporting program that captures > 99% of all births in the state [15]. Assuming at least 40% uptake of antenatal vaccines, a final sample of 450 respondents was required to estimate vaccine coverage with a precision +/− 4.5% at the standard 95% confidence interval. An initial sample size of 800 women was calculated after taking into consideration the proportion of women whom could be contacted by telephone in previous surveys of antenatal influenza vaccination uptake (~ 60%) and the participation rate among those contacted (> = 90%). Women selected at random from the MNS dataset were invited to participate via a letter sent from WA DOH and given the option to decline participation. The names and telephone number/s (as recorded in the MNS) of women who did not ‘opt-out’ were provided to WA DOH interviewers.

Participants were telephoned in December 2015 and asked to complete a 10-min telephone survey about whether they had been vaccinated against influenza and/or pertussis during their last pregnancy, and their reasons for being vaccinated/unvaccinated. Up to three telephone calls, at different times of day, were made to each woman; inability to make contact was recorded as ‘no response’. At the beginning of the telephone call, verbal consent was obtained to proceed with the interview and women who declined were not asked any further questions. Consent or declination was documented. Women who agreed to be interviewed were informed that they could cease the interview at any time. Information obtained during the interview included the mother’s age, education level, postcode of residence, the presence of chronic medical conditions, and the health care setting where the woman received the majority of her antenatal care. Because some women receive paper records of their vaccination, vaccinated women were asked for the date and batch number of the vaccine/s for verification purposes. Women who could not provide the batch number were asked for permission for WA DOH to retrieve details of the vaccination/s from their immunisation provider.

### Data analysis

Statistical analyses were performed using SAS version 9.4. To ensure representativeness of survey results, analyses were weighted by maternal age group and area of residence. Vaccination uptake of influenza, pertussis and both vaccines, along with 95% confidence intervals (CIs), were calculated. Univariate analysis was used to identify factors associated with vaccination uptake and variables significant at α = 0.05 were included in a hierarchical multivariate logistic regression model to control for potential confounding. Reasons for or against influenza or pertussis vaccination were compared using Pearson’s chi square analysis. Data from the 2015 antenatal survey were compared to results of published studies conducted in a similar manner during 2012–2014 [10, 16, 17].

This study has received written approval from the WA DOH Human Research Ethics Committee (HREC# 2015/29).

## Results

**Fig. 1:**
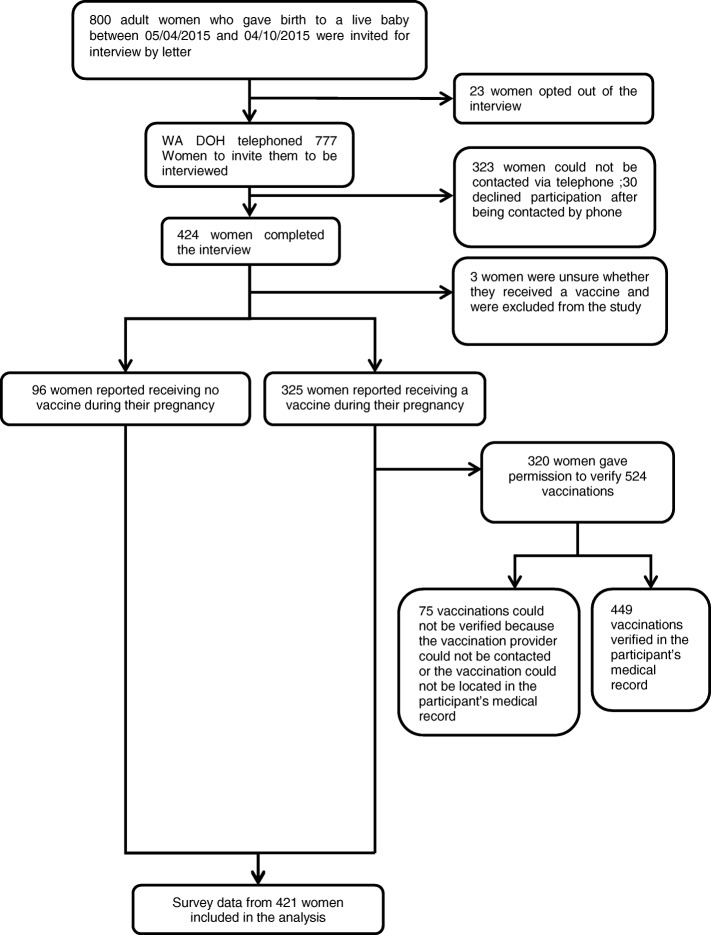
Participation in a telephone interview about vaccination during pregnancy – Western Australia 2015

Twenty-three (2.9%) of the 800 randomly selected women declined to participate in the study after receiving a letter informing them of their eligibility to participate; of the 777 remaining, 323 (41.6%) could not be contacted via telephone after 3 attempts, 30 (3.9%) declined participation after being contacted by telephone, and 424 (54.6%) completed the telephone survey (Fig. 1). Three women (3.9%) who were unsure whether they had received influenza or pertussis vaccine were excluded from the analysis.

The majority of survey participants (77.4%) lived in the Perth metropolitan area (Table 1); this is consistent with the proportion of births in the state in the Perth area (79.5%). However, slightly fewer participants were 18–24 years of age (10.9%) compared to all births during the study period (15.2%) and mothers 40 years and older were slightly over-represented (survey: 5.5%; state: 3.7%). Half of the women received most of their antenatal care at a public hospital antenatal clinic (49.6%), 30.4% from a private obstetrician, and 16.9% from a general practitioner. One in ten women (10.5%) had a chronic medical condition and about a third (31.3%) had a high school education or less.

**Table 1:** Results of univariate logistic regression analysis estimating the odds of pertussis and/or influenza vaccines during pregnancy – Western Australia, 2015

	Adult women who gave birth to a live infant in WA, 05/04/2015–04/10/2015	Survey respondents	Percent vaccinated against influenza^a^ (+/− pertussis vaccination)		Percent vaccinated against pertussis^c^ (+/− influenza vaccination)		Percent vaccinated against pertussis and influenza^d^	
	*n* (unweighted %)	*n* (unweighted %)	*n* (weighted %)	OR (95% CI)^b^	*n* (weighted %)	OR (95% CI) ^b^	*n* (weighted %)	OR (95% CI)^b^
Total	19,866	421 (100)	255 (60.6)		299 (71.0)	–	229 (54.4)	–
Age group								
18-24y	3010 (15.2)	46 (10.9)	32 (69.8)	Ref	33 (72.0)	Ref	28 (61.0)	Ref
25-29y	5713 (28.8)	109 (25.9)	61 (56.1)	0.55 (0.26–1.16)	71 (65.3)	0.73 (0.34–1.57)	55 (50.5)	0.55 (0.23–1.32)
30-34y	7049 (35.5)	164 (38.9)	98 (59.7)	0.64 (0.32–1.31)	124 (75.4)	1.20 (0.57–2.51)	89 (54.1)	0.90 (0.38–2.14)
35-39y	3368 (17.0)	79 (18.8)	54 (68.3)	0.94 (0.42–2.07)	57 (72.1)	1.01 (0.45–2.28)	48 (60.7)	0.95 (0.37–2.45)
≥ 40y	726 (3.7)	23 (5.5)	10 (43.5)	0.33 (0.12–0.95)	14 (60.9)	0.61 (0.21–1.75)	9 (39.1)	0.36 (0.10–1.21)
Residence								
Metropolitan	15,787 (79.5)	326 (77.4)	198 (60.9)	Ref	229 (70.2)	Ref	177 (54.4)	Ref
Non-metropolitan	4079 (20.5)	95 (22.6)	57 (60.6)	0.99 (0.61–1.60)	69 (72.9)	1.14 (0.67–1.64)	52 (55.00)	1.10 (0.60–2.00)
Educational attainment								
Primary/High School	–	132 (31.3)	79 (61.4)	Ref	92 (70.3)	Ref	72 (55.6)	Ref
TAFE^e^	–	98 (23.3)	59 (59.7)	0.93 (0.54–1.61)	66 (67.8)	0.89 (0.50–1.58)	52 (52.9)	0.89 (0.47–1.71)
University Undergraduate	–	102 (24.2)	63 (61.5)	1.00 (0.58–1.72)	77 (73.9)	1.20 (0.66–2.18)	56 (54.2)	1.24 (0.63–2.46)
University Postgraduate	–	89 (21.1)	54 (60.4)	0.96 (0.55–1.68)	64 (71.6)	1.07 (0.58–1.95)	49 (54.8)	1.04 (0.53–2.04)
Socioeconomic status^f^								
Quintile 1 and 2 (Lowest)	–	57 (13.5)	36 (63.0)	Ref	42 (72.7)	Ref	35 (61.0)	Ref
Quintile 3	–	117 (27.8)	66 (56.7)	0.77 (0.40–1.50)	72 (62.2)	0.59 (0.29–1.20)^h^	56 (48.6)	0.65 (0.30–1.41)
Quintile 4	–	89 (21.2)	52 (58.4)	0.83 (0.41–1.66)	66 (73.1)	0.97 (0.45–2.10)	48 (53.44)	0.97 (0.42–2.23)
Quintile 5 (Highest)	–	158 (37.5)	101 (64.5)	1.07 (0.56–2.03)	119 (75.0)	1.07 (0.53–2.17)	90 (57.2)	1.29 (0.60–2.77)
Chronic medical conditions^g^								
Yes	–	44 (10.5)	20 (48.9)	0.58 (0.30–1.10)	23 (52.8)	0.42 (0.22–0.80) ^h^	15 (35.9)	0.40 (0.18–0.86)^h^
No	–	377 (89.5)	235 (62.3)	Ref	276 (73.0)	Ref	214 (56.8)	Ref
Antenatal care provider								
Private Obstetrician	–	128 (30.4)	86 (66.6)	1.57 (0.98–2.51)	104 (81.1)	2.42 (1.41–4.13) ^h^	78 (60.3)	2.86 (1.51–5.42)^h^
General Practitioner	–	71 (16.9)	46 (65.5)	1.49 (0.84–2.65)	53 (75.3)	1.71 (0.92–3.18)	41 (59.1)	1.91 (0.93–3.92)
Private Practice Midwife	–	10 (2.4)	6 (67.7)	1.65 (0.44–6.14)	6 (67.7)	1.18 (0.32–4.39)	6 (67.7)	1.25 (0.33–4.70)
Public Hospital Antenatal Clinic	–	209 (49.6)	116 (56.0)	Ref	135 (64.1)	Ref	103 (49.3)	Ref
Other	–	3 (0.71)	1 (37.9)	0.48 (0.04–5.60)	1 (37.9)	0.34 (0.03–4.00)	1 (37.9)	0.362 (0.03–4.28)
Recommendation by healthcare provider								
Recommended pertussis only	–	37 (8.8)	10 (25.4)	1.56 (0.59–4.09)	26 (73.8)	5.34 (2.19–13.00)^h^	10 (25.4)	3.73 (1.23–11.35)^h^
Recommended influenza only	–	44 (10.5)	21 (49.3)	4.47 (1.89–10.59)^i^	15 (33.3)	0.94 (0.42–2.11)	10 (22.3)	2.16 (0.78–6.03)
Recommended pertussis and influenza vaccines	–	268 (63.7)	211 (79.4)	17.66 (8.92–34.99)^i^	231 (86.4)	11.96 (6.53–21.91)^i^	197 (74.3)	33.34 (15.15–73.38)^i^
Not recommended either vaccine	–	72 (17.1)	13 (17.9)	Ref	26 (34.6)	Ref	12 (16.7)	Ref

^a^Woman received seasonal influenza vaccine during pregnancy +/− pertussis vaccination

^b^Odds of vaccination and corresponding 95% confidence intervals

^c^Woman received diphtheria-tetanus-acellular pertussis vaccine during pregnancy +/− influenza vaccination

^d^Woman received both influenza and pertussis vaccines during pregnancy

^e^TAFE, technical and further education qualification

^f^Socioeconomic level was determined based on postcode of residence and Socioeconomic Index for Areas (insert link to website)

^g^Chronic medical conditions included asthma, chronic heart disease, chronic lung conditions, and diabetes

^h^p < .05

^i^p < .001

The proportions of women who reported that their health care provider recommended that they receive the influenza, pertussis, or both vaccines, were 74.0% (95% CI: 69.7–78.3%), 72.4% (95% CI: 68.0–76.7%) and 63.2% (95% CI: 58.5–67.9%), respectively. There were no differences in the sociodemographic characteristics of women who were recommended and those who were not recommended influenza and/or pertussis vaccines (*p* > .05). The proportions of women who reported they had received influenza, pertussis and both influenza and pertussis vaccinations during their last pregnancy were 60.6% (95% CI: 56.0–65.6%), 71.0% (95% CI: 66.3–75.2%) and 54.5% (95% CI: 49.7–59.4%), respectively.

**Fig. 2:**
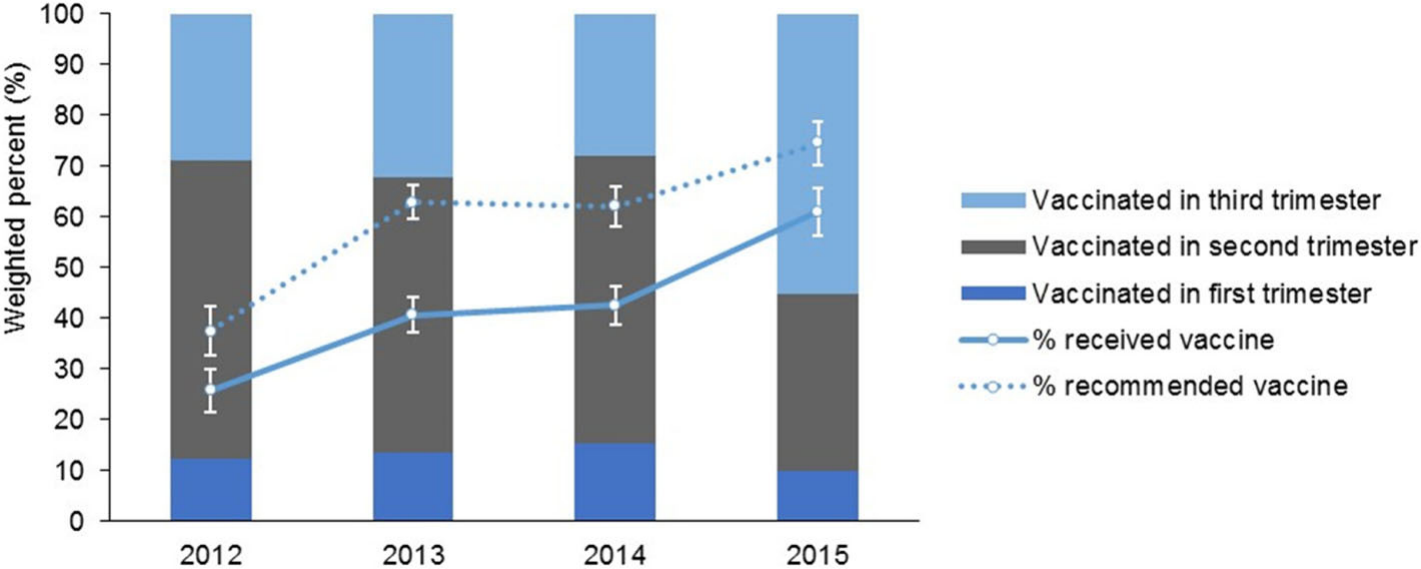
Trimester of antenatal influenza vaccination, and proportions of women who had been recommended vaccination and received vaccination, by year, Western Australia, 2012–2015

Influenza vaccine uptake increased significantly in 2015 with the annual antenatal vaccination survey from 2014 estimating coverage at 42.5% (95% CI: 38.8–46.3%). In addition, prior to 2015, the majority of women immunised against seasonal influenza received their vaccination in the second trimester (range: 54.3% [2013] to 58.9% [2012]); in 2015, this proportion declined to 28.1% while the proportion of immunised women who received their vaccination in the third trimester rose to 55.3% (Fig. 2). Most (90.1%) women immunised against pertussis received the vaccine in their third trimester and of the 211 women who received influenza and pertussis vaccine, 68.2% received both vaccines on the same day.

Of the 320 vaccinated women who gave permission for their immunisation record/s to be verified against medical records, 449 (85.7%) of the 524 reported vaccinations were confirmed (influenza: 79.6%, pertussis: 91.0%).

A total of 66.9% of women reported that they received their influenza vaccine at a general practice (GP), 17.9% at a public hospital antenatal clinic and 5.5% at their workplace; 68.6% of women reported receiving their pertussis vaccine at a GP, 20.9% at a public hospital antenatal clinic and 6.4% at a private hospital clinic.

### Predictors of vaccination

**Fig. 3:**
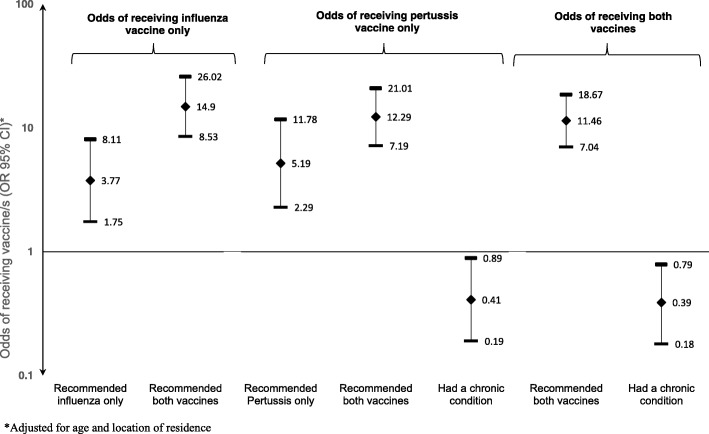
Multivariate logistic regression analysis of factors affecting antenatal pertussis and/or influenza uptake in Western Australia in 2015

On univariate analysis, a healthcare provider’s recommendation (*p* < .001) was significantly associated with the uptake of either influenza or pertussis vaccine during pregnancy (Table 1). The impact of the healthcare provider’s recommendation on vaccination appears to be vaccine specific, as women who were recommended pertussis vaccine (and not influenza vaccine) had a greater odds of pertussis (OR: 5.34, 95% CI: 1.23–13.00, *p* = 0.005) but not influenza, (OR: 1.56, 95% CI: 0.59–4.09, *p* = 0.37) vaccination. Similarly, women who were recommended influenza vaccine (and not pertussis vaccine) had greater odds of influenza (OR: 4.47, 95% CI: 1.89–10.59, *p* < 0.001) but not pertussis (OR: 0.94, 95% CI: 0.42–2.1, *p* = .89) vaccination compared to women not recommended to receive either vaccine. Women whose healthcare provider recommended both antenatal vaccinations had significantly higher odds of being vaccinated against both influenza and pertussis (OR 33.3, 95% CI: 15.15–73.38 *p* < 0.001). The existence of a chronic medical condition was negatively associated with pertussis vaccine uptake (OR 0.42, 95% CI: 0.22–0.80, *p* < 0.05) (Table 1). On multivariate analyses, a healthcare provider’s recommendation was the only common independent predictor of the uptake of influenza, pertussis and both vaccines (Fig. 3).

### Reasons for or against vaccination

Among vaccinated mothers, the most commonly reported reason they were immunised was to protect the baby (96.1% of mothers vaccinated against influenza and 98.6% of those vaccinated against pertussis). A significantly larger proportion of mothers vaccinated against pertussis vs influenza reported doing so because of influence of family, friends and media (73.7% vs 52.1%, *p* < 0.001) (Table 2).

**Table 2:** Reasons why women received/did not receive an influenza or pertussis vaccination – Western Australia, 2015 (multiple responses allowed)

Reasons why vaccinated women received a vaccine during pregnancy	Influenza vaccine (*n* = 256)	Pertussis vaccine (*n* = 299)	*p*-value
	*n* (%)	*n* (%)	
Protect baby	247 (96.1)	296 (98.6)	.30
Influenced by family, friends and media	136 (52.1)	222 (73.7)	<.001
Antenatal care provider recommended it	229 (90.6)	265 (88.4)	.48
General practitioner recommended it	155 (61.3)	172 (57.9)	.83
Worried about pertussis/influenza	138 (53.7)	188 (63.2)	.03
Obstetrician recommended it	129 (49.1)	157 (52.4)	.25
Midwife recommended it	128 (49.6)	165 (55.9)	.02
To protect family	6 (2.2)	–	–
To protect herself	11 (4.2)	–	–
Normally get vaccine	115 (44.6)	–	–
Health care employee	8 (2.9)	–	–
Chronic medical condition	16 (6.4)	–	–
Reasons why unvaccinated women did not receive a vaccine during pregnancy	Influenza vaccine (*n* = 165)	Pertussis vaccine (*n *= 122)	*p*-value
	*n* (%)	*n* (%)	
No antenatal care provider recommendation	56 (33.6)	54 (43.9)	.64
Worried that it would harm the baby	54 (32.5)	28 (23.0)	.47
Worried about potential side effects	62 (37.1)	15 (11.9)	.04
Was advised against it	11 (6.9)	8 (7.8)	.92
Was too late in pregnancy	–	7 (5.9)	–
Vaccine not available	6 (3.7)	3 (2.5)	.58
Already received or planning to receive after pregnancy	7 (6.9)	11 (8.6)	.10
Not necessary	6 (3.4)	–	–
Don’t normally get vaccine	56 (33.3)	–	–
First trimester of pregnancy	43 (25.8)	–	–

Commonly reported reasons for not being vaccinated against pertussis included that vaccination had not been recommended by an antenatal care provider (43.9%) and concerns about vaccination harming the baby (23.0%). Common reasons women did not receive influenza vaccine included concerns about side-effects to the mother (37.1%), harming the baby (32.5%) and because the vaccine was not recommended by a health provider (33.6%) (Table 2). Concern about the side effects of the vaccine were more commonly reported for influenza vaccine than pertussis vaccine (*p* = 0.04).

Among unvaccinated women, 53.6% (95% CI: 45.9–61.3) and 78.3% (95% CI: 70.4–85.3) reported that they would have been vaccinated against influenza and pertussis, respectively, during their pregnancy if a health care provider had recommended it.

## Discussion

This cross-sectional survey provides the first estimates of coverage and factors influencing uptake of both antenatal pertussis and influenza vaccines in Australia. A total of 72% of pregnant women received a pertussis vaccine; 61% received an influenza vaccine, an increase from 42% the previous year [16]. These results demonstrate that most women receive routinely recommended vaccines during pregnancy in Western Australia, but there is still room for improvement.

The introduction of the antenatal pertussis vaccination program in 2015 may have influenced seasonal influenza vaccination of pregnant women in terms of both uptake and trimester of vaccine administration. In contrast to previous years, 2015 was the first year that most women vaccinated against influenza received the vaccine in their third, rather than second trimester. As nearly 70% of women who vaccinated against both influenza and pertussis received them on the same day, it would seem that introduction of a recommendation for pertussis vaccination between weeks 28–32 of pregnancy may have had the effect of shifting the timing of the influenza vaccination to the third trimester as well as increasing the coverage of antenatal influenza vaccination. While vaccinating for influenza during the third trimester of pregnancy is ideal for antibody transfer [1] to the unborn child, it leaves pregnant women potentially unprotected against influenza during their first two trimesters of pregnancy. This may have serious adverse consequences for women at high risk of developing complications of influenza.

WA’s antenatal pertussis vaccination program was quite successful in its first year, given that in other settings less than 25% of women received a pertussis vaccine during pregnancy in the first year of their program [18, 19]. A recent study from the Northern Territory, Australia, found that 22.3% of women received a pertussis vaccination during pregnancy [18]. In the United Kingdom, the antenatal pertussis vaccination program was implemented for 4 years before a comparable coverage of antenatal pertussis vaccination was achieved (70%) [20]. Unpublished data from the WA Department of Health indicates that antenatal pertussis coverage in WA has not only been sustained, but has continued to increase to almost 80% in 2016.

One factor which may have influenced this success in WA is the potential influence of the tragic death of a young infant in early March 2015. At that time the mother was pregnant, antenatal pertussis vaccination was not recommended in the Australian Immunisation Handbook and there was no government-funded pertussis vaccination program in WA in place [21]. The baby’s death was well publicised and his family continue to promote the benefits of antenatal and childhood vaccination in Australia via mass- and social-media and parent and baby expos. The impact of their efforts is likely reflected in the high proportion of mothers who said they were vaccinated against pertussis because of the influence of family, friends and media (74%). This finding suggests that social-media and community-driven campaigns can be effective in promoting vaccinations among pregnant women.

Despite the success of WA’s antenatal pertussis vaccination program and continued increases in antenatal influenza uptake, further improvement in uptake is achievable and should be pursued. Results from this survey and other studies have consistently identified the recommendation by a healthcare provider as the strongest predictor of antenatal vaccination [12–14, 16–18]. Although influenza and pertussis vaccination were standard antenatal care for women in our study, less than two-thirds were recommended both vaccines during their pregnancy. Data from unvaccinated women in this survey suggest that if 100% of women were recommended to be vaccinated in accordance with current standard-of-care obstetrical guidelines in Australia, coverage rates among pregnant women for influenza and pertussis vaccine could reach 82% and 94%, respectively.

Barriers to vaccination reported by the women in this survey reveal a need for additional education for pregnant mothers and their antenatal care providers. Over a third of women not vaccinated for influenza and 27% of women not vaccinated for pertussis cited concerns about side effects of the vaccination to themselves or harm to their babies as reasons for non-vaccination. Other reasons reported for not vaccinating include already being immunised for pertussis before pregnancy and/or plans to vaccinate post-partum. None of these reasons for not being vaccinated in pregnancy are evidence-based decisions [3, 8, 10, 22]. The results also suggest that further education would be beneficial for antenatal care providers given that 8% and 7% of women not vaccinated for pertussis and influenza respectively reported that a healthcare provider had advised them against vaccination.

A negative association between having a chronic medical condition and pertussis vaccination uptake even after controlling for healthcare provider’s recommendation was unexpected. It is not clear why women with a chronic medical condition would be more likely than women without a chronic medical condition to refuse pertussis vaccination if it was offered.

There are several limitations to our study. First, assignment of vaccination status relied on self-report. Previous research has shown that vaccination coverage can be over-estimated based on self-report [23]. However, we were able to verify 86% of self-reported vaccinations directly with the immunisation provider, suggesting any bias introduced is likely to be small. Second, although women were selected at random to participate in the survey, there was some under-representation of mothers under the age of 25 years. To account for this under-representation, survey results were weighted by age and apart from this particular subset of women, age and geographic distribution of survey respondents was generally comparable with the population of women eligible for study selection. The response rate of 54.6% is considered satisfactory for a telephone survey [24]. Finally, this survey was conducted in WA and the views and opinions of mothers in this state may not represent those in other parts of Australia or other countries. Further assessments on the uptake of pertussis and influenza vaccines in other geographic settings are needed.

## Conclusions

Almost three-quarters of pregnant women were immunised in the first year of an antenatal pertussis vaccination program. Although increasing, antenatal influenza vaccine coverage remains lower than that for pertussis vaccine. A substantial proportion of unimmunised women indicated that they would have been vaccinated if it had been recommended to them by an antenatal care provider, suggesting that antenatal vaccination coverage approaching 90% could be achieved if providers universally recommended immunisation. Strategies for improving antenatal vaccination uptake should include education of pregnant women and their healthcare providers on the benefits and safety of influenza and pertussis vaccination during pregnancy.

## Abbreviations

CI: Confidence interval
GP: General Practitioner
MNS: Midwives Notification System
WA DOH: Western Australian Department of Health
WA: Western Australia
